# Modulation of STAT6 signaling for hepatoprotection 

**DOI:** 10.3389/fphar.2025.1659227

**Published:** 2025-12-02

**Authors:** Emmanuel Somm, Mahdi Rahman, Ildiko Szanto, Karim Gariani, François R. Jornayvaz

**Affiliations:** 1 Service of Endocrinology, Diabetes and Metabolism, Department of Medicine, Geneva University Hospitals/University of Geneva, Geneva, Switzerland; 2 Diabetes Center, Faculty of Medicine, University of Geneva, Geneva, Switzerland; 3 Department of Cell Physiology and Metabolism, University of Geneva, Geneva, Switzerland

**Keywords:** STAT6, Ischemia-reperfusion, acute liver damage, MASLD, MASH, fibrosis, HCC, immune polarization

## Abstract

Signal transducer and activator of transcription (STAT) proteins are a family of seven transcription factors mediating various biological processes. STAT6 is classically known to regulate immune cell biology by transmitting signals from interleukin (IL)-4 and IL-13 into transcriptional activation of genes driving type 2 immunity. In orchestrating T helper lymphocytes and macrophages polarization, STAT6 plays a central role in the regulation of both cellular and humoral immunities. Several pathologies, including inflammatory disorders, autoimmune/allergic diseases, metabolic syndrome as well as cancer, are associated with a dysregulation of type 2 immunity related to inadequate expression and/or activity of STAT6. In the present review, following a brief introduction of STAT6 biology, we summarize the immunologic and physiological roles of STAT6 in the context of liver integrity as well as the potential roles of STAT6-mediated pathways in both hepatoprotection and liver pathophysiological mechanisms.

## 1 Introduction

Signal transducer and activator of transcription (STAT) proteins are a family of seven transcription factors (STAT1, STAT2, STAT3, STAT4, STAT5A, STAT5B and STAT6) involved in pleiotropic biological processes such as cell proliferation, apoptosis, differentiation and immunity (Darnell et al., 1994). STAT proteins are activated through phosphorylation in the cytoplasm by Janus Kinases (JAKs), a group of tyrosine kinases associated with receptors of different ligand classes, mainly cytokines or growth factors (Schindler and Darnell, 1995; Wang and Levy, 2012). One of the main functions of STAT proteins is the modulation of immune system reactions by transmitting signals from cytokine receptors and inducing transcriptional activation of genes involved in humoral and cellular immunity (Goenka and Kaplan, 2011; Hou et al., 1994). Depending on the pathogens and cytokine environment produced by other immune cells, the naïve T helper (Th) lymphocytes (Th0) cells undergo differentiation towards a Th1 or Th2 phenotype (Butcher and Zhu, 2021). Th1 lineage cells will produce pro-inflammatory cytokines such as Tumor Necrosis Factor alpha (TNF-α), interleukin (IL)-1 and interferon (IFN)-γ (Zhu and Paul, 2008). These pro-inflammatory cytokines activate cellular immunity by stimulating macrophages, natural killer cells and CD8^+^ cytotoxic T cells (Zhu and Paul, 2008). In contrast, Th2 differentiation is triggered by eosinophils, basophils and mast cells initially producing IL-4, resulting in the secretion of anti-inflammatory cytokines such as IL-4, IL-5, and IL-13 (Tolomeo and Cascio, 2024). These interleukins also induce the synthesis of antigen-specific antibodies in B cells (humoral response) (Takeda et al., 1997). STAT6 is activated by the binding of IL-4 and IL-13 to their cognate receptors and thus induces Th2 immune response (Hou et al., 1994). Adequate balance between Th1 and Th2 lymphocytes and cytokines is primordial for both the efficiency and harmlessness of the immune system. Several pathologies, including inflammatory or autoimmune disorders, allergic diseases, as well as cancer, are associated with dysregulated Th1/Th2 equilibrium and related to inadequate expression/activity of STAT6 (Tolomeo and Cascio, 2024). In consequence, STAT6 represents a main driver of the adaptive immune system.

**FIGURE 1:**
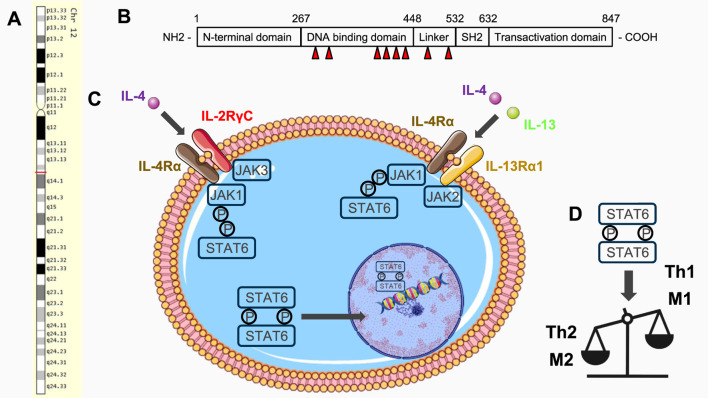
**(A)** Chromosomal location of STAT6 gene on chromosome 12q13.3-q14.1 in humans (Source: https://www.genecards.org). **(B)** Structure of STAT6 protein. Location of somatic mutations identified in humans are indicated by red arrows. Numbers refer to amino acids. **(C)** Representation of STAT6 signaling pathway. Illustrations have been created manually using PowerPoint (Microsoft) and images originating from Servier Medical Art (https://smart.servier.com/), licensed under CC BY 4.0 (https://creativecommons.org/licenses/by/4.0/). **(D)** Phosphorylated STAT6 promotes type 2 immunity including type 2 T helper and M2 macrophage polarization.

The STAT6 gene consists of 23 exons located on chromosome 12q13.3-q14.1 in humans (Figure 1A) encoding a 94 kDa protein composed of 847 amino acids (Figure 1B). Of note, the homolog STAT6b presents an NH2-terminal truncation while the homolog STAT6c presents a SH2 domain deletion (Tolomeo and Cascio, 2024; Hebenstreit et al., 2006). STAT6 and STAT6b are phosphorylated on tyrosine residue in response to IL-4/IL-4 receptor interaction while STAT6c is not and thus lacks the capacity to induce cell proliferation (Tolomeo and Cascio, 2024; Hebenstreit et al., 2006). A shorter isoform of STAT6 is also specifically present in mast cells (Tolomeo and Cascio, 2024; Hebenstreit et al., 2006). As the other members of the STAT family, STAT6 contains six domains: (1) a helical N-terminal domain (ND) responsible for interactions between STAT dimers and DNA, (2) a coiled-coil (CC) domain binding regulatory factors, (3) a DNA-binding domain (DBD) binding enhancers of the GAS family, (4) a helical linker (LK) domain involved in nuclear translocation and DNA binding, (5) a Src homology 2 (SH2) domain binding cytokine receptor following tyrosine phosphorylation and (6) a C-terminal transactivation domain (TAD) triggering transcription of targeted genes (Tolomeo and Cascio, 2024; Hebenstreit et al., 2006) (Figure 1B).

Mechanistically, IL-4 binds first to the IL-4 receptor α-chain (IL4Rα) which then recruits either the IL-2Rγc or the IL-13Rα1 (Tolomeo and Cascio, 2024; Hebenstreit et al., 2006; Murata et al., 1999; Junttila, 2018) (Figure 1C). Binding of the IL-4/IL-4Rα complex to IL-2Rγc or IL-13Rα1 is required for the constitution of a functional heterodimer receptor complex, inducing a conformational change in the intracellular receptor domains that lead to the phosphorylation of the Jak kinases associated with IL-4Rα (Jak1), γc (Jak3), or IL-13Rα1 (Jak2 also named Tyk2) (Tolomeo and Cascio, 2024; Hebenstreit et al., 2006; Murata et al., 1999; Junttila, 2018) (Figure 1C). IL-2Rγc is generally expressed in lymphocytes while IL-13Rα1 is mostly expressed in non-hematopoietic cells (Tolomeo and Cascio, 2024). The tyrosine residues in the intracellular domains of IL-4Rα act as docking sites for the SH2 domain of STAT6, resulting in homodimerization and nuclear translocation of STAT6 thereafter binding specific DNA motives in diverse transcription regulatory regions of target genes (Tolomeo and Cascio, 2024; Hebenstreit et al., 2006; Murata et al., 1999; Junttila, 2018) (Figure 1C). Similarly, IL-13 binds IL-13Rα1 and the complex recruits IL-4Rα leading to analog activation of STAT6 (Tolomeo and Cascio, 2024; Hebenstreit et al., 2006; Murata et al., 1999; Junttila, 2018) (Figure 1C). STAT6 mainly acts as an activator of transcription but it could also acts as a repressor of transcription (Elo et al., 2010) or through other mechanisms including binding of transcriptional cofactors or epigenetic modification (Ohmori and Hamilton, 2000). In addition, STAT6 also presents post-translational modifications, such as phosphorylation, ubiquitination, adenosine diphosphate (ADP)-ribosylation and acetylation that can be targeted to develop therapeutic strategies (Huang et al., 2020).

Signaling mediated by STAT6 is required for the Th2 immune response at different levels. Firstly, STAT6 is involved in Th2 cell differentiation through a feed-forward mechanism implicating the Th2 master switch GATA-binding protein 3 (GATA3) (Scheinman and Avni, 2009). Consequently, STAT6 deficiency decreases the number of cells harboring the Th2 phenotype (Shimoda et al., 1996; Takeda et al., 1996). Secondly, STAT6 increases the pool of Th2 cells by increasing their proliferation while preventing their apoptosis through the independent growth factor-1 (Gfi-1) (Kaplan et al., 1998). STAT6 also impacts B cells in switching their immunoglobulin class (Shimoda et al., 1996). Accordingly, STAT6-deficient mice have impaired circulating immunoglobulin (Ig)E and IgG1 in response to conventional T-dependent antigens (Linehan et al., 1998) while in human, IL-4 induces B lymphocytes switch from the expression of IgM to the expression of IgG1, IgG4 and IgE (Tolomeo et al., 2022; Stavnezer, 1996). STAT6 also increases B cells’ expression of cell surface molecules including MHC class II molecules, IL-4Rα, CD80, CD86, and CD23 (Bruns et al., 2003). In addition, STAT6 inhibits B cells apoptosis by increasing the expression of Bcl-xL, which suppresses the mitochondrial apoptotic pathway (Wurster et al., 2002).

STAT6 is also a key player in macrophage activation. In fact, macrophages can oscillate between two states of polarization: the classical M1 activated state, featured by the production of pro-inflammatory cytokines, and the M2 alternatively activated state characterized by the production of anti-inflammatory cytokines. M1 activation is classically triggered by IFN-γ and toll-like receptor ligands while M2 activation is induced by IL-4 and IL-13 that activate STAT6 signaling (Sica and Mantovani, 2012). In macrophages, IL-4/STAT6 signaling also increases the activity of peroxisome proliferator-activated receptor γ (PPARγ), a transcription factor regulating both lipid metabolism and macrophage activity (Szanto et al., 2010). In addition, IL-4/STAT6 signaling also induces PPARγ-coactivator-1β (PGC-1β), that drives oxidative metabolism, and which could act as a co-activator of STAT6 in polarization of M2 macrophages (Vats et al., 2006).

Beyond its nuclear function, STAT6 is also associated with mitochondria in human hepatocytes, as well as endothelial and vascular smooth muscle cells (Khan et al., 2013). STAT6 possesses mitochondrial-targeting sequences and transmembrane segments anchored to the outer membrane of mitochondria (Kim et al., 2022). In this context, STAT6 interacts with mitofusin 2, inhibiting mitochondrial fusion. Mitochondrial STAT6-mitofusin two interaction can be induced by hypoxia, resulting in mitochondrial fragmentation, cytochrome c release and apoptosis (Kim et al., 2022). Moreover, IL-13/STAT6 signaling can increase mitochondrial reactive oxygen species production and decrease the mitochondrial membrane potential and ATP levels, leading to mitochondrial dysfunction and cellular senescence (Zhu et al., 2022).

Compared to wild-type (WT) mice, STAT6-deficient mice are resistant to allergic airway inflammation (Akimoto et al., 1998). On the other hand, genetically engineered mouse models with increased STAT6 activity present an exacerbated allergic inflammation (Bruns et al., 2003; Daniel et al., 2000). While human STAT6 deficiency has not yet been identified, as it may not be viable, human STAT6 single nucleotide polymorphisms are associated with multiple allergic and non-allergic diseases (Sharma et al., 2023), including atopic dermatitis, multiple food allergies, anaphylaxis, asthma, allergic rhinitis, eosinophilic gastrointestinal diseases, lymphoproliferation, osteoporosis, cerebral aneurysms, renal fibrosis, short stature, and hypotrichosis (for review see (Tolomeo and Cascio, 2024; Sturvey and Consortium, 2024)).

**FIGURE 2:**
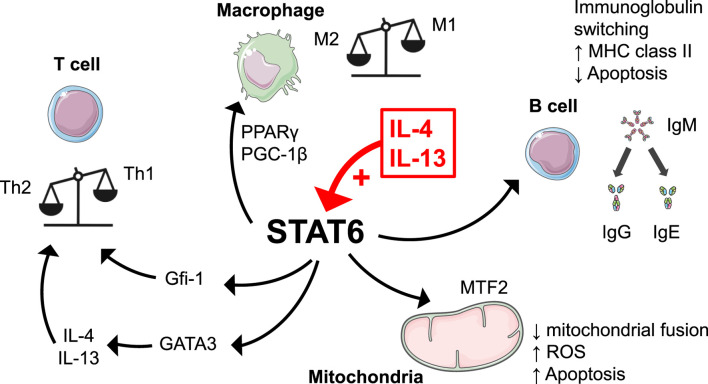
Stat6 signaling mediates pleiotropic immune and cellular process. STAT6 is implicated in Th2 immune response through the transcription factors GATA3 and Gfi-1. STAT6 also promote M2 (alternative) macrophage polarization through metabolic regulators PPARγ and PGC-1β. STAT6 acts on B cells, switching their immunoglobulin class, increasing the expression of various cell surface markers and inhibiting their apoptosis. STAT6 can also be anchored in mitochondrial membrane to interact with mitofusin 2, inhibiting mitochondrial fusion and triggering apoptosis and cellular senescence. Illustrations have been created manually using PowerPoint (Microsoft) and images originating from Servier Medical Art (https://smart.servier.com/), licensed under CC BY 4.0 (https://creativecommons.org/licenses/by/4.0/).

Pleiotropic immune and cellular process driven by STAT6 are summarized in Figure 2.

In the present review, we summarize and briefly discuss the immunologic and physiological roles of STAT6 in the context of liver integrity and the potential role of this signaling pathway in both hepatoprotection and liver pathophysiological mechanisms.

## 2 Role of STAT6 in acute liver injury context

### 2.1 Ischemia/reperfusion (I/R)

Ischemia/reperfusion (I/R) injury represents an important physiological challenge, notably in the clinical context of organ transplantation. One of the components linked to hepatic I/R damage are activated CD4^+^ cells. A first study reported that following I/R, STAT6-deficient mice present identical hepatocellular damage and neutrophil accumulation compared to WT mice, in contrast to STAT4 deficient mice and nu/nu mice harboring a T cell deficiency that display reduced liver injury (Shen et al., 2003). Another publication has shown that contrary to WT mice, STAT6-deficient mice treated with recombinant adenovirus encoding IL-13 failed to improve hepatic function/histology during I/R injury, suggesting a mitigating effect for STAT6 in the inflammatory I/R response (Ke et al., 2004). In line with these data, administration of IL-13 reduced the production of pro-inflammatory proteins, suppressed liver neutrophil recruitment, hepatocellular injury and liver edema independently of NF-κB activation but greatly increased the activation of STAT6 (Yoshidome et al., 1999). In addition, IL-4 treatment protected liver grafts from transplantation-related I/R damage by polarizing Kupffer cells towards the anti-inflammatory M2 phenotype via the STAT6-JMJD3 pathway (Deng et al., 2020).

In the post-ischemic state, the acidic microenvironment resulting from increased anaerobic glycolysis promoted M1 but inhibited M2 polarization of macrophages and PPAR-γ signaling (Ding et al., 2021). Accordingly, the PPAR-γ agonist GW1929 inhibited M1 polarization and reduced I/R under acidic environment, representing an interesting therapeutic option in this context (Ding et al., 2021). Similarly, injection of mesenchymal stem cells prior to hepatic warm I/R restrained M1 but boosted M2 polarization of Kupffer cells via enhanced STAT6 phosphorylation, contributing to liver regeneration in fulminant hepatic failure in mice (Shang et al., 2023). Interestingly, deletion of the cell division cycle 42 (Cdc42) protein in myeloid cells alleviated hepatic necrosis and inflammation in I/R by favoring M2 polarization of hepatic myeloid macrophages via STAT6 activation (He et al., 2024). In line with these findings, the Cdc42 inhibitor ML141 protected mice from hepatic I/R injury (He et al., 2024).

### 2.2 Acute liver failure (ALF)

ALF is an inflammatory liver condition with high mortality. M2 macrophages, infiltrating the liver, play an important role in the prevention of ALF-related hepatocyte injury. In this context, it has been shown that mesenchymal stem cells alleviate ALF through STAT6-mediated M2 macrophage polarization (Li et al., 2021). The therapeutic potential of mesenchymal stem cells on ALF seems also to be dependent on the secretion of prostaglandin E_2_ (PGE_2_). In fact, mesenchymal stem cells-derived PGE_2_ inhibited NLR Family Pyrin Domain Containing 3 (NLRP3) inflammasome activity and its subsequent production of inflammatory cytokines, leading to M2 macrophage differentiation (Wang J. et al., 2021). In addition, mesenchymal stem cells treatment improved liver function by directly promoting M2 macrophage polarization via the JAK1/STAT6 signaling pathway in mouse models of ALF (Li Z. H. et al., 2023). Analogously, chemical compounds, such as the active halophenol derivative 2,4′,5′-Trihydroxyl-5,2′-dibromo diphenylmethanone (LM49), also attenuated ALF via the activation of the JAK1/STAT6 signaling pathway (Yang et al., 2021).

Taken together, the previously mentioned works suggest a protection mediated by STAT6 and its primordial role in macrophage polarization in the specific contexts of I/R and ALF. In contrast, in other biological contexts, and particularly in case of chronic hepatic damage, several other works highlight deleterious roles for STAT6-mediated immunity.

### 2.3 Concanavalin A (ConA)-induced liver injury

Con A is a plant lectin extracted from jack beans that binds to the mannose residues of several glycoproteins. As a translational model, ConA activates lymphocytes and when administered to mice, induces liver injury triggered by macrophage-mediated activation of T lymphocytes (Tiegs et al., 1992). In this context, STAT6 is rapidly activated under ConA administration (Jaruga et al., 2003). Accordingly, STAT6-deficient mice present abolished ConA-mediated liver injury with no change in IFN-γ/STAT1, IL-6/STAT3 or TNF-α/NF-κB signaling or natural killer T (NKT) cells activation (Jaruga et al., 2003). Mechanistically, infiltration of neutrophils and eosinophils in ConA-induced hepatitis is inhibited in STAT6-deficient mice compared to WT mice (Jaruga et al., 2003), suggesting that STAT6 plays a critical role in ConA-induced hepatitis. ConA interferes with Protein kinase C (PKC) localization and activity (Costa-Casnellie et al., 1985), (Matsumoto et al., 1993). Thus, PKC-zeta deficient mice display mitigated ConA-induced inflammation and reduced hepatocellular damage in parallel with the ablation of STAT6 tyrosine phosphorylation (Duran et al., 2004). In agreement with this deleterious action of STAT6 in hepatocyte survival, activation of Jak1/STAT6 signaling induces eotaxin in hepatocytes and triggers IL-5 production in NKT cells, both pathways promoting liver eosinophil recruitment and damage (Moscat et al., 2006). Moreover, IL-4 induces apoptosis of human hepatocytes through STAT6 activation in association with a decrease in mitochondrial membrane potential and an increase in caspase activation, independently of the Fas pathway (Aoudjehane et al., 2007). Of note, on the opposite to ConA, various phytochemicals (Chinese herbs, herb formulas) favorably regulate the STAT6 signaling pathway (Chen et al., 2022).

### 2.4 Parasitism

*Schistosoma* is a trematode that invades through the skin, affecting over 200 million people worldwide. Resistance to schistosoma infection is associated with a strong Th2 immune response in humans, which could lead to liver damage if not controlled. Interestingly, polymorphism (rs324013) in the STAT6 gene acts synergistically with IL-13 polymorphism (rs1800925) in human susceptibility to schistosomiasis (Isnard and Chevillard, 2008). Moreover, in C57BL/6 mice infected with *Schistosoma japonicum*, liver fibrosis is associated with enhanced phosphorylation of STAT6, in accordance with the hepatic upregulation of IL-4 and IL-13 receptors (Duan et al., 2019). Of note, egg-derived extracellular vesicles from *Schistosoma japonicum* contain Sja-miR-71a microRNA (miR) that inhibits both IL-13/STAT6 and Transforming growth factor (TGF)-β1/SMAD pathways via direct targeting of semaphorin 4D, leading to suppression of liver fibrosis by regulating the Th1/Th2/Th17/Treg balance (Wang et al., 2020).

**FIGURE 3:**
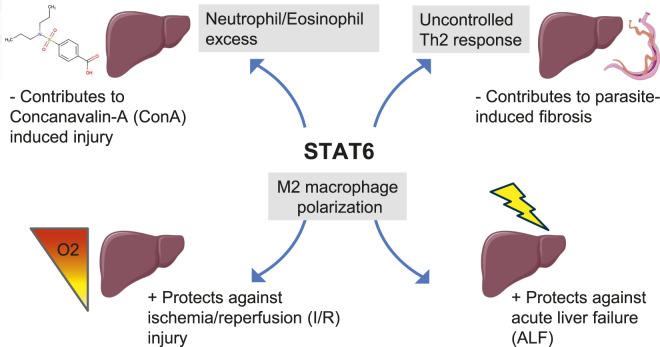
STAT6 is mechanistically involved in various models of acute hepatic disorders. Illustrations have been created manually using PowerPoint (Microsoft) and images originating from Servier Medical Art (https://smart.servier.com/), licensed under CC BY 4.0 (https://creativecommons.org/licenses/by/4.0/).

Figure 3 illustrates the experimental situations of acute hepatic disorders in which STAT6 has been implicated and Table 1 described experimental details of studies investigating the role of STAT6 in this context.

**TABLE 1:** Detailed descriptions of the experimental studies (including design, duration, and treatment details) investigating the role of STAT6 in acute liver injury context.

	Experimental / model design	Animal detail / treatment	Ref
I/R	Partial lobar warm ischemia model (90 min)/6 h of reperfusion	STAT4 and STAT6 KO mice	Shen et al. (2003)
	Partial lobar warm ischemia model (90 min)/6 h of reperfusion	STAT6 KO mice	Ke et al. (2004)
	Partial lobar warm ischemia model (90 min)/4 h of reperfusion	WT mice treated with 1 μg of rIL-13 via the lateral tail vein before ischemia	Yoshidome et al. (1999)
	Liver grafts preserved for 18 h (at 4°C) prior to liver transplantation Tissue analysis 6 h after transplantation	Rats infused with rIL-4 (5 ng/kg/min via portal vein) / Clodronate liposome (50 mg/kg) 24 h before transplantation or GSK-J4 (5 mg/kg) 3 days before transplantation	Deng et al. (2020)
	Clamp of the left/middle branches of portal vein and hepatic artery for 60 min (70% of liver ischemia) / Various reperfusion time	Mice receiving clodronate liposomes (200 μl) through tail vein	Ding et al. (2021)
	Clamp of the hepatic artery, portal vein and bile duct branches to the left and median liver lobes for 60 min / 6 h of reperfusion	Mesenchymal stem cells suspension was injected 30 min prior I/R	Shang et al. (2023)
	1 h ischemia / 12 h reperfusion	Myeloid Cdc42 KO mice	He et al. (2024)
ALF	Rats injected i.p. with D-galactosamine (1.2 g/kg)	i.v. transplantation with 5.5 × 10^5^ mesenchymal stem cells 12 h after	Li et al. (2021)
	Mice injected i.p. with 600 mg/kg D-galactosamine and 100 μg/kg LPS/ 6 h after, i.v. transplantation with 2×10^6^ mesenchymal stem cell	TAK1 inhibitor 5z-7-ox (5 mg/kg) or EP4-specific antagonist (GW627368X, 20 mg/kg) initiated 1 h before LPS/D-Galactosamine treatment	Wang et al. (2021a)
	Mice injected with 10% carbon tetrachloride (5 mL/kg) twice a week for 8 weeks + a single dose (8 mL/kg) 3 days later	Transplantation with 1 × 10^5^ mesenchymal stem cell via tail vein immediately after carbon tetrachloride initiation	Li et al. (2023a)
	Acute liver injury modeled by a single i.p. LPS injection (10 mg/kg b.w.) / Mice were analyzed 24 h thereafter	Different concentrations of LM49 (4.5-40.5 mg/kg) and Levofloxacin were administrated intragastrically each day 3 days before LPS treatment	Yang et al. (2021)
Concanavalin A	Mice were injected i.v. with concanavalin A (12–20 μg/g) / Mice were analyzed within 24 h postinjection	WT mice, STAT6 and IL-4 KO mice	Jaruga et al. (2003)
	Mice were injected i.v. with concanavalin A (12–20 μg/g) / Mice were analyzed within 24 h postinjection	WT and ζPKC KO mice treated with 100 ng of murine IL-4	Duran et al. (2004)
Schistosoma infection	Hairless abdomen skin was exposed to Schistosoma japonicum cercariae for 15-20 min / Mice were analyzed 6-10 weeks after infection	WT mice	Duan et al. (2019)

## 3 Role of STAT6 in chronic liver injury context

### 3.1 MASLD/MASH and lipid metabolism

Metabolic dysfunction-associated steatotic liver disease/steatohepatitis (MASLD/MASH) is currently the most common hepatic disorder in industrialized countries, mainly due to the obesity and type 2 diabetes pandemic (Younossi, 2019). MASLD can evolve towards a state of hepatic inflammation (steatohepatitis/MASH) (Younossi, 2019). Lifestyle changes could have beneficial effects on hepatic steatosis, but efficient drugs to limit progression of MASLD/MASH are still lacking (Younossi, 2019).

The first study investigating the implication of STAT6 signaling on liver homeostasis during MASLD/MASH analyzed the hepatic proteome of STAT6-deficient mice on chow and high-fat diet (HFD) using liquid chromatography–mass spectrometry (LC-MS) (Iff et al., 2009). In this study, changes in protein content indicated a disturbed lipid homeostasis and a state of hepatocellular stress in STAT6-deficient mice (Iff et al., 2009). Notably, hepatic fatty acid binding protein 1 (FABP1) was increased concomitantly to increased steatosis in STAT6 deficiency (Iff et al., 2009). Accordingly, another study demonstrated that exogenous IL-25 administration protects against hepatic steatosis through IL-13-induced activation of STAT6 (Wang et al., 2015). IL-25 promotes hepatic macrophage differentiation towards the M2a phenotype, both *in vivo* and *in vitro*, via the IL-13/STAT6 pathway, alleviating HFD-induced hepatic steatosis (Zheng et al., 2019).

Mechanistically, several works have highlighted the role of the IL-4/STAT6 immune axis on peripheral nutrient metabolism, notably through interactions between STAT6 and the nuclear receptor family of PPARs.

In specific, STAT6 interacts with PPAR-γ to elicit macrophage polarization towards an anti-inflammatory/insulin-sensitizing phenotype (Szanto et al., 2010). Moreover, liver proteome analysis of WT and STAT6-deficient mice treated with the PPAR-γ agonist rosiglitazone has shown that STAT6 modulate the expression of pyruvate kinase M2 (PKM2), an enzyme involved in the control of glycolysis and cell proliferation (Sajic et al., 2013). Interestingly, rosiglitazone induced PKM2 in liver but repressed its expression in adipose tissue. In addition, rosiglitazone limited liver steatosis while enhancing adipose fat accumulation and insulin sensitivity in STAT6-deficient mice (Sajic et al., 2013), suggesting a complex interaction between STAT6 and PPAR-γ in the regulation of whole-body fat distribution.

STAT6 also inhibits cholesterol synthesis through the miR-197–forkhead box protein J2 (FOXJ2) axis (Dubey and Saini, 2015). In addition, activation of STAT6 by IL-4 enhances insulin action by inhibiting the PPAR-α driven nutrient catabolism and adipose tissue inflammation (Ricardo-Gonzalez et al., 2010), illustrating molecular crosstalk between the immune system and macronutrient metabolism. Interestingly, the isoflavone formononetin ameliorated hepatocyte apoptosis, inflammatory response, and liver dysfunction through upregulation of STAT6 phosphorylation and downregulation of Protein Tyrosine Phosphatase 1B (PTP1B). PTP1B diminishes STAT6 signaling by dephosphorylating its S325 residue in its DNA binding domain and also acts as a negative regulator of the insulin signaling pathway by dephosphorylating the Tyr 1162 and 1163 residues of the insulin receptor (Wang et al., 2024).

STAT6-mediated processes are involved in the modulation of diverse other aspects of macrophage differentiation. For example, deletion of Inositol requiring enzyme 1α (IRE1α) (a marker of endoplasmic reticulum stress) could activate STAT6 and shift macrophages polarization towards the M2/anti-inflammatory state (Yang et al., 2018). In addition, mice with myeloid cell-specific deficiency for the transcription factor FoxO1 are protected against diet-induced MASH, revealing that FoxO1 counteracts STAT6 resulting in an increased number of macrophages differentiating towards the M1 state (Lee et al., 2022). STAT6 also interacts with PPAR-α-mediated effects. Indeed, data obtained from the Gene Expression Omnibus (GEO) and the BXD mouse reference population demonstrated that the Th2 cytokines IL-4 and IL-13 increase the secretion of the hepato-protector fibroblast growth factor 21 (FGF21) in the liver in a STAT6-dependent manner through PPAR-α inhibition (Kang et al., 2021).

STAT6 has also been involved in bile homeostasis. In fact, STAT6 phosphorylation by IL-4 or IL-13 increases the expression of Anoctamin-1 (TMEM16A), the Ca^2+^-activated Cl^−^ channel in cholangiocytes, which contributes to ductular bile formation (Dutta et al., 2020). This function of STAT6 in bile formation/secretion makes STAT6 a potential target in cholestatic liver disorders. In accordance, dilauroylphosphatidylcholine activates liver receptor homolog-1 (LRH-1) which in turn induces phosphorylation and transcriptional activity of STAT6, and thus, promoting M2 macrophage polarization. This signaling cascade prevents liver injury and cholestasis (Ghos et al., 2024). In addition, the JAK1/2 inhibitor ruxolitinib reduces portal inflammation and bile duct damage in humans (Shao et al., 2022), inhibits the signaling of IFN-γ and the secretion of pro-inflammatory cytokines (IL-6, TNF-α and MCP-1) and promotes a STAT6-dependent macrophage polarization in the context of autoimmune cholangitis. Moreover, in murine liver, natural type 2 innate lymphoid cells (ILC2s) undergo expansion and increase amphiregulin production to drive STAT6-dependent epithelial proliferation (Russi et al., 2023). In line with these data, ILC2 transcripts are positively associated with cholangiocyte abundance in patients suffering from biliary atresia (Russi et al., 2023).

### 3.2 Fibrosis

While liver fat storage and inflammation are quite correctable, fibrosis resulting from these insults is often considered as a much less reversible step in the progression of liver disease. It has been shown that the IL-4/STAT6 and IL-13/STAT6 signaling pathways exacerbate the progression of metabolically induced liver fibrosis in mice on HFD (Hart et al., 2017). Accordingly, IL-13 serum levels as well as the IL-13 hepatic transcript content are elevated in patients with MASH compared to controls (Shimamura et al., 2008; Weng et al., 2009). Several lines of evidence suggest that STAT6 is involved in homeostasis and functioning of cell types implicated in extracellular matrix remodeling and collagen production. In fact, in culture of human liver myofibroblasts, STAT6 was activated by IL-4 and increased production of collagens I, III and IV (Aoudjehane et al., 2008). IL-4 and IL-13 induce miR-142-5p in macrophages sustaining their profibrogenic action (Su et al., 2015). Accordingly, *in vitro*, miR-142-5p mimics prolonged STAT6 phosphorylation by targeting its negative regulator SOCS1 (Su et al., 2015).

Hepatic stellate cells (HSCs) represent the main source of hepatic collagen production during MASLD/MASH. In the HSC cell line LI90, that expresses IL-4 and IL-13 receptors, as well as phosphorylated STAT6*, in vitro* administration of IL-4 or IL-13 increased the production of collagen while suppressing cell proliferation (Sugimoto et al., 2005). In human activated HSCs obtained from MASH biopsies, gene expression of IL-13Rα2 is upregulated (Shimamura et al., 2008) and STAT6-mediated HSC activation is triggered by IL-13 secreted by ILC2 cells (McHedlidze et al., 2013). However, IL-13 can also induce the profibrogenic connective tissue growth factor (CTGF) production by HSCs in damaged liver independently of STAT6 phosphorylation (Liu et al., 2011). In addition to its transactivating activity, STAT6 seems to directly interact with several proteins to trigger fibrosis. Accordingly, a protein-protein complex consisting of TGF-β1 receptor, Glutamyl-prolyl-tRNA synthetase (EPRS), Janus kinases, and STAT6 mediates prolyl-transfer RNA synthetase (PRS)-driven fibrosis (Song et al., 2019). Accordingly, the selective prolyl-tRNA synthetase (PRS) inhibitor (DWN12088) inhibits pro-fibrotic gene expression by suppressing TGFβR1/glutamyl-prolyl-tRNA synthetase (EPRS)/STAT6 axis signaling in the context of diet-induced MASH/fibrosis (Lee et al., 2024).

STAT6 has also been implicated in the fibrotic process occurring in response to carbon tetrachloride (CCl4) exposure. Of note, pro-fibrotic genes expressions are positively correlated with STAT6 activation in the liver of mice treated with CCl4 (Song et al., 2019). Interestingly, nutritional interventions modulating STAT6 signaling present anti-fibrotic properties. Oral administration of a bioactive chitooligosaccharide limits liver fibrosis in CCl4-exposed mice through mechanisms implicating the JAK1/STAT6 pathway in M2 macrophages/Kupffer cells (Liu et al., 2022). In addition, the traditional Chinese medicine Qijia Rougan Formula mitigated extracellular matrix deposition and fibrosis in the liver of CCl4-exposed rats by inhibiting macrophage M2 polarization (Zheng et al., 2023). Similarly, the monoterpenoid glycoside Paeoniflorin inhibits hepatic stellate cell activation and alleviate CCl4-induced extracellular matrix deposition via JAK2/STAT6 inhibition (Ma et al., 2020).

## 4 Role of STAT6 in liver cancer

STAT proteins not only orchestrate immune cell pools and activity but can also impact tumor cells. In fact, STAT proteins shape distinct metabolic/energetic processes that regulate tumor progression and even therapy resistance by transducing signals from metabolites, cytokines and growth factors (Li Y. J. et al., 2023).

Solitary fibrous tumor are rare fibroblastic mesenchymal tumors that can occur at virtually any site within the body (Thway et al., 2016). Despite the benign character of the tumor, 15%–20% of patients progress with either local recurrence or distant metastases (Tariq et al., 2021; Davanzo et al., 2018). One of the proteins that is linked to this tumor development is the NGFI-A Binding 11 Protein 2 (NAB2). NAB2 typically acts as a repressor of early growth response zinc finger DNA transcription factors. Patients suffering from solitary fibrous tumor and hemangiopericytomas present an intrachromosomal fusion between STAT6 and NAB2 genes, leading to the constitutive activation of NAB2 (Singh et al., 2021; Robinson et al., 2013; Chmielecki et al., 2013).

Beyond this case study, several evidence involve STAT6-driven type 2 polarization of immunity in cancer. In tumor micro-environment, different infiltrated cells can promote tumor growth and invasiveness, including M2 tumor-associated macrophages (TAMs). In fact, M2 macrophage polarization is involved in the inflammatory processes of breast (Rahal et al., 2018), colorectal (Chen et al., 2016), and lung (Fu et al., 2019) malignancies. In line with these studies, STAT6 pharmacologic inhibitors reduced tumor growth and metastatic process in both breast and gastric cancer through modulation of macrophage M2 polarization (Binnemars-Postma et al., 2018; Lu et al., 2018).

Analogously, several works have delineated a role for STAT6 in the development and metastasis of hepatocellular carcinoma (HCC), the most common type of primary liver cancer.

First, *in vitro* works have confirmed the role of STAT6 in cell cycle maintenance. STAT6 silencing significantly inhibited HepG2 and Hep3B hepatoma cells survival and proliferation (Qing et al., 2017). In agreement, nuclear expression of the metalloreductase STEAP3 significantly stimulated HCC cells proliferation by promoting cell cycle progression via a STAT6/Rac Family Small GTPase 1 (RAC1)/JNK signaling axis (Wang L. L. et al., 2021). Exposure of HuH7 and Hep3B hepatoma cells to IFN-α or IFN-β led to the formation of STAT2/STAT6 complexes, triggering the secretion of the anti-inflammatory interleukin-1 receptor antagonist (IL-1Ra) (Wan et al., 2008). Similarly, the administration of STAT6 inhibitor AS1517499 significantly attenuated tumor growth and early liver metastasis in an orthotopic 4T1 mammary carcinoma mouse model (Binnemars-Postma et al., 2018). STAT6 inhibitor treatment suppressed the M2 polarization and exerted an anti-HCC effect (Kong and Guo, 2023).

In addition, STAT6 induces the expression of the pyruvate kinase M2 (PKM2), an enzyme regulating both glycolysis and proliferation (Sajic et al., 2013). In this way, a STAT6 inhibition that dampens the expression of PKM2 could suppress the growth of tumor cells that are highly dependent of glycolysis. In fact, PKM2 activation promotes metastasis of HCC and inhibition of tumor cell autophagy (Yu et al., 2021; Park et al., 2016).

Another factor influencing the oncogenic role of STAT6 involves various long non-coding RNAs. Different long non-coding RNAs (lncRNAs) regulate STAT6 signaling with potential implication in liver oncogenesis. In the macrophage THP-1 cell line co-cultured with the liver cancer cell line H22, lncRNA-Colorectal Neoplasia Differentially Expressed (CRNDE) overexpression leads to STAT6 upregulation (Han et al., 2021). *In vivo*, downregulation of CRNDE mitigated tumor volume, diminished the expression of key angiogenesis-related proteins and simultaneously suppressed the expression of STAT6 and its phosphorylation. CRNDE could indirectly regulate tumor angiogenesis by promoting M2 polarization of macrophages, which is also one of the mechanisms of microenvironmental immune regulation in liver cancer (Han et al., 2021). Human and mouse Kupffer cells from metabolically induced HCC displayed increased lncRNA SNHG20 expression compared with MASLD Kupffer cells (Wang B. et al., 2019). In addition, lncRNA SNHG20 overexpression induced M2 polarization through STAT6 activation, while SNHG20 silencing concomitantly delayed STAT6-dependent M2 polarization and the progression of MASLD to HCC in mice (Wang B. et al., 2019).

After removal of the primary tumor, STAT6-deficient mice rejected liver metastasis and lived longer than WT mice in the same conditions (Ostrand-Rosenberg et al., 2002). STAT6 deficiency also corrected liver injury and inflammation induced by alpha-galactosylceramide, a specific agonist for invariant natural killer T (iNKT) cells evaluated in the context of treatment for liver cancer (Wang et al., 2013). Of note, STAT6 deficiency in Scurfy (sf) mice lacking Treg cells shortened their lifespan and increased their hepatic inflammation, suggesting a protective role of STAT6 in case of Treg cell depletion (Suscovich et al., 2012).

Anti-tumoral actions of different molecules/treatments have been associated with modulation of STAT6 signaling. For example, betulinic acid inhibits STAT6 phosphorylation and decreases M2 polarization in the microenvironment of liver cancer, resulting in antitumoral effect (Guo et al., 2023). Like the STAT6 inhibitor, the multi tyrosine kinase receptor inhibitor sunitinib suppressed M2 polarization of RAW264.7 murine macrophages and diminished JAK1-STAT6 signaling both *in vitro* and *in vivo* in mice, leading to dampening of the malignant behaviors of HCC cells (Kong and Guo, 2023). This anti-HCC action of sunitinib is related to its suppressive effect on the expression of Ki67 (Kong and Guo, 2023). More surprisingly, low-inorganic phosphate stress irreversibly repolarized tumor-associated macrophages towards the M1 phenotype by silencing STAT6 and activating the p65 subunit of NFκB (Lv et al., 2023). The major vault protein significantly increased infiltration of M2-type tumor-associated macrophages in tumor tissues of HCC patients, promoting HCC proliferation, metastasis, and invasion through enhanced STAT6 activity (Yu et al., 2023). In addition, in myofibroblasts the myeloid differentiation primary response protein 88 (MyD88) can promote MASLD-induced hepatocarcinogenesis by enhancing macrophage M2 polarization through a mechanism involving the C-C chemokine receptor type 1 (CCR1) receptor and the STAT6/PPAR-β pathway (Liu et al., 2024).

Clinically, analysis of a cohort containing hepatitis B virus-infected HCC patients (GSE14520) and data from The Cancer Genome Atlas showed that elevated STAT6 expression is a prognostic biomarker for HCC (Wang X. et al., 2019). Accordingly, bioinformatic analyses confirmed enrichment of STAT6 in pathways involved in cell cycle, cell division and lipid metabolism (Wang X. et al., 2019). Of note, STAT6 has been shown to be differentially expressed in tumor and non-tumor tissues (Wang X. et al., 2019). Moreover, STAT6 predicts a worse prognosis in HCC patients (Qing et al., 2017) and the overexpression of STAT6 was markedly correlated with more advanced clinical stages and pathological grades in HCC (Qi et al., 2020). Interestingly, STAT6 might be related to the gender prevalence of HCC. In fact, estrogen suppressed tumor growth functions by inhibiting the JAK1/STAT6 signaling pathway that drives macrophage M2 activation (Yan et al., 2012), potentially explaining predominance of HCC in men compared to women. In tumor-associated macrophages originating from HCC patients, membrane transport proteins responsible for the absorption of zinc (*Zip9*) promoted STAT6 phosphorylation and M2 macrophage polarization and concomitant inhibition of M1 macrophage polarization (Gou et al., 2022).

**FIGURE 4:**
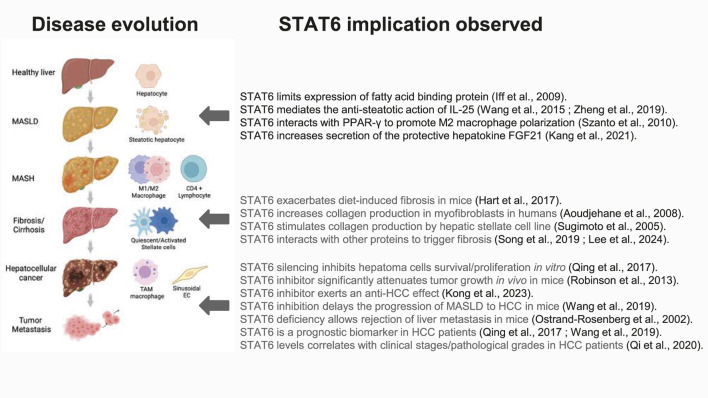
STAT6-related effects in MASLD/MASH/HCC progression. Illustrations have been created manually using PowerPoint (Microsoft) and images originating from Biorender (https://www.biorender.com/).

Figure 4 illustrates the pathophysiological steps leading to MASLD/MASH and HCC progression that are associated to STAT6 activity or that can be targeted by STAT6 modulators and Table 2 described experimental details of studies investigating the role of STAT6 in this context.

**TABLE 2:** Detailed descriptions of experimental studies (including design, duration, and treatment details) investigating the role of STAT6 in chronic liver injury context.

	Experimental/model design	Animal detail/treatment	Ref
MASLD/MASH	Animals were kept on a high‐fat diet (60% kcal from fat) for 10 weeks	WT and STAT6 KO mice	Iff et al. (2009)
	Animals kept under chow diet	STAT6 KO mice treated with rosiglitazone i.p. (10 mg/kg b.w.) for 10 days	Sajic et al. (2013)
	Mice placed on a HFD for 15 weeks	PPARα, STAT6 KO mice. Treatment with IL‐4 (2 μg) twice a week for 8 weeks	Ricardo‐Gonzalez et al. (2010)
	Mice placed on a HFD+0.5 % cholesterol for 8 weeks followed by i.p. administration of streptozotocin (45 mg/kg) for 3 days	Oral administration of formononetin (100 mg/kg per day for 4 weeks)	Wang et al. (2024)
	Animals kept under chow diet.	STAT6 KO mice injected i.p. with rIL-4 (2 μg/mouse) or rIL-33 (0.5 μg/mouse)	Kang et al. (2021)
	Mice placed on chow diet.	IL-4RA, STAT6 and IL-13 KO mice. Daily i.p. IL-33 injected (1 μg) for 4 days	Russi et al. (2023)
Fibrosis	Mice were fed a HFD (60% kcal from fat) for 15–40 weeks or the AMLN diet (40% fat/20% fructose/2% cholesterol) for 15 weeks	IL-4, IL-10, IL-12, IFN-γ KO mice. 250 μg of anti–TGF-β or anti-IL-13 antibody was injected i.p. twice weekly	Hart et al. (2017)
	Injection with CCl_4_i.p. (1 mg/kg) once a week for 5 weeks	WT and*Eprs*haploinsuffisent mice	Song et al. (2019)
	Mice on a methionine-choline deficient (MCD)-diet for 15 weeks	Oral gavage of WT mice for 6 weeks with DWN12088 (10 mg/kg)	Lee et al. (2024)
	Mice were treated i.p. with 20% CCl_4_solution at a dosage of 4 mL/kg twice weekly for 4 weeks	WT mice were treated orally with different concentrations (100–250 mg/kg daily) of chitooligosaccharide once a day for 4 weeks	Liu et al. (2022)
	s.c. injection of CCl_4_ (40%, 2 mL/kg)	Rats daily treated with Qijia Rougan Formula (10 ml/kg) for 6 weeks	Zheng et al. (2023)
Cancer	Female mice were injected with 4T1-luc cells (1 × 10^5^) into the mammary fat pad and tumors were allowed to develop	Treatment with AS1517499 (20 mg/kg, i.p. twice per week) was started when the tumor volume reached a volume of ±100 mm^3^	Binnemars-Postma et al. (2018)
	0.2 mL cell suspension (containing 6 × 10^6^ cells) was injected to mice. 3–4 days later, nodular tissue appeared at the injection site	Nude mice were given intragastric administration of Sunitinib (0.5–1 mg/kg) once daily for 25 consecutive days	Kong and Guo (2023)
	injection of 0.2 mL (5 × 10^7^/ml) of H22 cells cell suspension into nude mice. Visible subcutaneous solid tumors appeared around 10–13 days	Mice were injected i.v. with CRNDE antisense nucleotides (10 mg/kg) once a week for 4 consecutive weeks or with the pcDNA3.1‐CRNDE plasmid (15 μg)	Han et al. (2021)
	Mice were fed with HFD (8–36 weeks) and injected i.p. with diethyl nitrosamine (45 mg/kg) once a week for a total of 20 weeks	WT mice were injected with LV-sh-SNHG20-infected RAW264.7 cells through the tail vein	Wang et al. (2019a)
	4T1 mammary carcinoma cells were injected into the mammary gland	Mice treated with IL-13Rα2-Fc (0.2 mg/200 μL/dose) each day for 14 days	Ostrand-Rosenberg et al. (2002)
	i.v. injection of a single α-Galcer (3 μg) was administered	WT, IFN-γ, IFNGR, IL-4, IL-4Ra, STAT1 and STAT6 KO mice were used	Wang et al. (2013)
	Injection of 5 × 10^6^ cells suspension. Nodular tissue 1 week after	Nude mice daily treated with betulinic (10–20 mg/kg) orally for 25 days	Guo et al. (2023)
	Mice were injected subcutaneously with 1 × 10^6^ Hepa1‐6 cells	WT mice were injected with 1 mg sevelamer every day for 14 days	Lv et al. (2023)
	Mice were injected s.c. with Hepa1-6 cells (1.5-3.0 × 10^6^)	Mice injected i.p. with clodronate liposomes (200 μL/mouse) every 3 days	Yu et al. (2023)
	Mice injected i.p. with DEN (50 μg/g b.w. at 15 days) + 10 months HFD. Mice were s.c. injected with Hepa1-6 cells (1 × 10^6^ cells)	WT and MyD88 KO mice were injected i.p. with 3 mg/kg of CCR1 inhibitor J113863 twice a week for 2 weeks	Liu et al. (2024)

## 5 Conclusion

Several pathophysiological conditions are associated with an imbalance in immune cells polarization linked to inadequate STAT6 signaling. In consequence, STAT6 could represent an interesting therapeutic target, notably in the field of liver disorders. STAT6 appears to have dual roles. In fact, STAT6 presents a protective role in limiting inflammatory I/R response and acute liver failure. An activation of STAT6 could present a valuable interest in these pathophysiological contexts. In contrast, STAT6 activation appears detrimental in cases of fibrosis and liver tumors. Accordingly, pharmacological inhibitors or specific antisense oligonucleotides inhibiting STAT6 have shown interesting properties *in vitro* as well as in animal models to limit liver fibrosis or HCC occurrence/progression. Other related drugs also interfering with STAT6 signaling, such as the selective inhibitor of EGFR tyrosine kinase domain Gefitinib, also inhibiting IL-13/STAT6, could present important benefits, notably to enhance immunosurveillance in an oncologic field.
